# Bone sialoprotein: a multifunctional regulator of bone remodelling and tumour progression

**DOI:** 10.1038/s41413-025-00490-5

**Published:** 2026-01-19

**Authors:** Valentina Kottmann, Philipp Drees, Erol Gercek, Ulrike Ritz

**Affiliations:** Department of Orthopaedics and Traumatology, University Medical Center, Johannes Gutenberg University, Mainz, Germany

**Keywords:** Bone cancer, Bone

## Abstract

Bone sialoprotein (BSP) is a major non-collagenous protein of the bone extracellular matrix and an important regulator of bone formation and resorption. BSP is produced by bone cells and chondrocytes and present in the bone matrix, cells, dentin and cartilage. However, its aberrant expression in primary tumour tissues and the sera of cancer patients with metastases implicates BSP in tumour biology and progression. The Arg-Gly-Asp (RGD) motif of BSP may be crucial not only for the attachment of metastasising cells to the bone surface but also for tumour growth, survival and activity. This review examines the structure and functions of BSP, including its roles in angiogenesis, bone formation, osteoclast differentiation and activity and cancer cell proliferation, survival, complement evasion, adhesion, migration and invasion. Growing evidence highlights BSP as a key mediator of tumour pathophysiology, skeletal metastasis development and associated bone remodelling. These processes are driven through RGD-integrin binding, the integrin/BSP/matrix metalloproteinase axis, integrin-independent signalling pathways, epithelial-to-mesenchymal transition and potentially post-translational modifications. A deeper understanding of BSP’s role in tumour progression may reinforce its potential as a prognostic and diagnostic tumour biomarker and aid the development of anti-BSP antibodies or targeted inhibitors for skeletal metastases and bone diseases.

## Introduction

Bone sialoprotein (BSP) is a non-collagenous protein of the extracellular matrix (ECM) of mineralised tissues, playing a crucial role in bone formation, bone resorption and bone remodelling. As a member of the SIBLING (Small Integrin-Binding Ligand, N-linked Glycoprotein) family, BSP is involved in processes of cell adhesion, osteoclast and osteoblast differentiation, angiogenesis and tumour progression. Its regulatory functions in bone metabolism and cancer metastasis, make it an interesting target for scientific studies. This review explores the structure, function and clinical relevance of BSP, highlighting its importance in skeletal biology and disease progression.

## Bone sialoprotein

BSP, also known as integrin-binding sialoprotein (IBSP) or bone sialoprotein-2 (BSP2), belongs to the SIBLING family. This family includes four additional proteins: osteopontin (OPN), dentin matrix protein 1 (DMP-1), dentin sialophosphoprotein (DSSP) and matrix extracellular phosphoglycoprotein (MEPE).^1^ In human, all SIBLING genes are located within a 375 kb region on chromosome 4q21,^2^ whereas the BSP gene in mice is found in the long arm of chromosome 5 (5q).^3^ Although the intrinsic sequences of the SIBLING proteins are not very homologous, they share similar exon structures and a conserved arginine–glycine–aspartic acid (RGD) sequence, which mediates cell attachment and signalling.^2^ SIBLING proteins are primarily expressed in bone and dentin, where they are secreted into the unmineralised ECM, known as osteoid and the bone tissue is mineralised.^4,5^ Their activity and function, including those of BSP, are influenced by post-translational modifications (PTMs) such as glycosylation, phosphorylation and sulphation.^6^

### Gene structure of bone sialoprotein

**Fig. 1:**
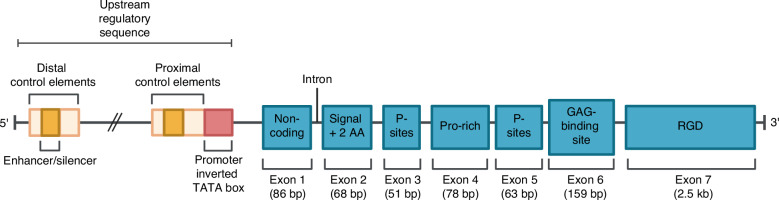
Human bone sialoprotein (BSP) gene structure with the proximal and distal promoter. AA amino acids, GAG glycosaminoglycan P phosphorylation, Pro proline, RGD arginine–glycine–aspartic acid. Modified from Bouleftour et al.^9^ and Ogata et al.^8^ Created with BioRender.com

BSP is encoded by the 15-kb IBSP gene located on chromosome 4q21.1,^7^ which consists of seven exons separated by six introns. Exon 1 of 82 base pairs (bp) is non-coding, while exon 2 (68 bp) encodes the signal peptide and the initial two amino acids of the mature protein. Exon 3 (51 bp) and exon 5 (63 bp) encode casein kinase II phosphorylation sites. Exon 4 (78 bp) encodes proline-rich regions,^8^ and exon 6 (159 bp) encodes a glycosaminoglycan binding domain.^9^ Exon 7 (2.5 kb) encodes over 50% of the protein, including the RGD sequence. Polyglutamic acid residues are present in exons 5 and 7.^10^ The gene structure is shown in Fig. 1.

The human BSP promoter contains an inverted CCAAT-box (ATTGG) (bp −54 to −50) and an inverted TATA-box (TTTATA) (bp −28 to −23), which are highly conserved among species.^11^ The proximal BSP promoter contains two cyclic AMP response elements (CREs), CRE1 (−79 to −72) and CRE2 (−674 to −667),^12–14^ along with a potential runt-related transcription factor 2 (Runx2) binding site (−110 bp),^15^ a fibroblast growth factor 2 (FGF2) response element (FRE) (−96 to −89),^12,16^ an activator protein (AP)1 element (−148 to −142),^10^ a homeobox (HOX) binding site (−200 to −191),^10^ and a shear stress response element (SSRE)1 (−893 to –888).^17^ Located at the distal human BSP promoter is a SMAD family member 4 (SMAD4) binding site (−1 874 to −1 867)^18^ and a SSRE2 (−2 474 to −2 469).^17^ Human BSP promoter may contain additional AP1 response elements and an AP2 response element. AP1(2) element is found at positions −483 to −477.^12^ The location of AP1(3) is approximately within −800 to −791.^12,17^ AP2 element is located at the distal human BSP promoter (−1 148 to −1 139).^17^

Several transcription factors regulate IBSP expression by bindings its promoter, including CRE binding protein 1 (CREB1), phosphorylated CREB1 (phosphor-CREB1), Runx2, Msh homeobox 2 (Msx2), distal-less homeobox 5 (Dlx5), SMAD1 and the AP-1 family (c-Fos, c-Jun, JunD and Fra2).^15,17,19,20^ The co-activator p300 also interacts with these factors to enhance IBSP transcription.^19^

### Protein structure of bone sialoprotein

This section provides an overview of the protein structure of BSP. The potential roles and relevance of specific regions, binding motifs and PTMs are discussed in more detail later in the context of BSP functions in bone remodelling, cancer progression and immune evasion.

Native human BSP consists of 317 amino acids, including a 16-amino acid signal peptide.^21^ The core protein has a molecular weight of 33.6 kD^22^; however, PTMs and a low isoelectric point (pKi) increase its molecular weight to ~70–80 kD.^23,24^ PTMs include N- and O-linked glycosylation, serine (Ser)/threonine (Thr) phosphorylation and tyrosine (Tyr) sulphation. Human BSP contains three polyglutamic acid rich sequences: one near the N-terminus (residues 52–87) and two repeats in the middle of the protein (residues 130–157 and 179–188),^25^ comprising around 22% of the total amino acids.^26^ BSP’s high acidity is attributed to phosphorylation, sulphation and its polyglutamic acid content. BSP can be divided into three domains: an amino-terminal (residues 1–100), a central (residues 100–250) and a carboxyl-terminal domain (residues 250–317).

The N-terminal region is rich in Tyr.^27^ It includes a seven-amino-acid sequence L-H-R-R-V-K-I (leucine-histidine-arginine-arginine-lysine-valine-isoleucine) that binds to heparin and heparan sulphate proteoglycans,^21^ potentially enhancing RGD-integrin interactions.^28^ The murine analogue is F-H-R-R-I-K-A (phenylalanine-histidine-arginine-arginine-isoleucine-lysine-alanine).^28^ Additionally, a collagen (COL)-binding site is located near the N-terminus,^29^ with residues 19-46 in rat BSP shown to bind to COL independently of PTMs.^30^ The COL-binding region is implicated in angiogenesis, bone formation, and cancer cell adhesion (see sections on BSP functions for further details). The N-terminal and central domains mediate hydroxyapatite (HA) nucleation and binding. These polyglutamic acid-rich regions^25^ are implicated in osteoblast differentiation,^31^ mineralisation,^27,31^ and may influence osteoclast activity.^32^ In rat and porcine BSP, these domains nucleate and bind HA.^33–36^ Specifically, in porcine BSP, residues 42–125 and 133–272 can nucleate HA independent of the intact protein structure.^37^ The C-terminal domain contains the RGD motif,^9^ a key regulator of cell chemotaxis and integrin-mediated signalling,^38,39^ which is involved in skeletal biology and cancer. Adhesive capacity of human BSP depends on native protein structure, as denaturation reduces cell adhesion.^24^ Additionally, two Tyr-rich regions, located on either side of the RGD sequence, may further support BSP-mediated cell adhesion.^40^ Research suggests that Tyr297 or Tyr298 may undergo sulphation.^22^

Both the amino-terminal and central domains are highly glycosylated. The predicted N-linked glycosylation sites are the asparagine (Asn) residues at positions 88, 161, 166 and 174,^24^ with Asn161 and Asn166 being the most probable.^22^ The O-linked glycosylation sites are predominantly situated between Thr213 and Ser232.^22^ Glycosylation may significantly affect the activity and function of BSP. In bovine BSP, heat-desialylation enhances COL binding compared to untreated protein.^41^ In contrast, glycosylation does not appear to influence HA nucleation and formation in porcine BSP.^37^ Overall, human BSP is heavily glycosylated, containing 33.8% oligosaccharides, with 64% being O-linked and 36% N-linked.^22^ Phosphorylation also modulates function.

In bovine BSP, phosphorylation of Thr residues near the RGD sequence reduces rat R1 fibroblast adhesion,^42^ while dephosphorylation reduces receptor activator of NF-κB ligand (RANKL) expression and parathyroid hormone (PTH)-stimulated bone resorption in primary mouse bone marrow macrophages (BMMs), implicating this PTM in osteoclast regulation.^43^ Effects on HA nucleation vary: in porcine BSP, dephosphorylation has no impact,^34^ whereas phosphorylation of Ser136 in rat BSP—located close to polyglutamic acid repeats—promotes HA nucleation.^44^ Carboxylation is also critical for HA nucleation in porcine BSP.^34^ PTMs collectively account for ~30%–40% of human BSP’s total molecular weight.^24^

**Fig. 2:**
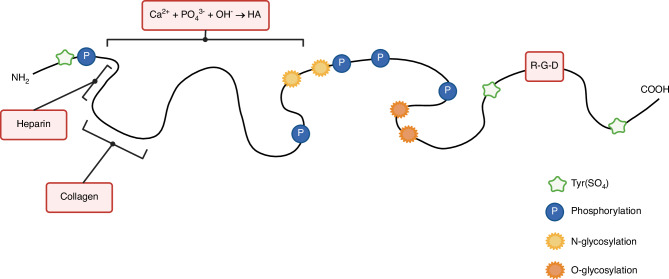
Unfolded protein structure of human bone sialoprotein (BSP). HA hydroxyapatite, RGD arginine–glycine–aspartic acid, Tyr(SO_4_) tyrosine sulphation. Modified from Bouleftour et al.^9^ and Ganss et al.^174^ Created with BioRender.com

BSP’s open, flexible structure allows the interaction with various binding partners.^42,45^ Its secondary structure has a low α-helix content (5%), a high β-sheet content (32%) and a significant amount of random coil (46%).^24^ The high β-sheet content may support BSP’s role in matrix mineralisation and in mediating cell-matrix and cell-cell interactions. The random coil regions could undergo folding upon binding to ligands such as integrins, COL, or heparin, although the functional significance of these secondary structures remains to be further characterised. A simple schematic model of the unfolded human BSP is shown in Fig. 2.

### Regulation of bone sialoprotein expression

BSP is produced by various cell types including osteoblasts,^4,46^ osteoclasts,^4^ osteocytes,^4,46^ bone marrow stromal cells (BMSCs),^47^ and chondrocytes.^4^ Platelets^48^ and placental trophoblast cells^4^ also express BSP mRNA. Notably, BSP is also synthesised by tumour cells in both primary tumours^49^ and breast cancer bone metastases.^50^

#### Hormonal regulation of bone sialoprotein expression

PTH (human 1–34 PTH) increases BSP mRNA and protein expression in human osteoblast-like SaOs-2 cells (10 nmol/L, 3 h) via Tyr kinase and PKA pathways, and increased interactions between CRE1/2 and CRE-binding proteins.^12^ Similarly, PTH(1-34) stimulates BSP transcription in embryonic chicken osteoblasts (10 nmol/L, 8 h) through cAMP/PKA-mediated CRE activation.^51^ In contrast, parathyroid hormone-related protein (PTHrP) [human PTHrP (1–34)] decreases BSP mRNA via the same pathway in murine cementoblast-like cells (100 nmol/L, 6 and 24 h).^52^ In rat osteoblast-like ROS 17/2.8 cells, oestrogen receptor alpha (ERα) overexpression promotes BSP gene expression via CRE, AP-1 and glucocorticoid response element (GRE) promoter elements.^53^ Calcitriol (1,25-dihydroxyvitamin D3), the active form of vitamin D, downregulates BSP gene expression in rat bone organ cultures (10 nmol/L, 5 days).^54^

#### Regulation of bone sialoprotein expression by growth factors and cytokines

Interleukin 8 (IL-8) (10 ng/mL recombinant human IL-8, 48 h) stimulates BSP mRNA and protein in human LNCaP and DU145 prostate adenocarcinoma (pAdC) cells.^55^ IL-11 (20 ng/mL) induces BSP gene expression in SaOs-2 (6 h) and rat ROS17/2.8 (12 h) cells via Tyr kinase, ERK1/2 and PI3K/Akt signalling,^14,56^ and enhanced CREB1, phospho-CREB1 and AP-1 (c-Fos, c-Jun) interactions.^14^

FGF-2 (10 ng/mL, 3 and 12 h) enhances BSP gene expression in SaOs-2 cells by promoting nuclear protein binding to FRE and AP1 elements.^20^ In DU-145 cells, FGF-2 (10 ng/mL) and forskolin (FSK) (1 μmol/L), an inducer of intracellular cAMP formation, upregulate BSP mRNA and protein expression via cAMP/FGF2 activation, and targeting the CRE1/2 promoter elements (3 and 12 h).^19^ Additionally, FGF-2 (10 ng/mL) and FSK (1 μmol/L) stimulate PKA, Tyr kinase and MAPK signalling pathways and have been shown to engage with the inverted CCAAT box (ATTGG), and the BSP promoter elements CRE, FRE and pituitary-specific transcription factor-1 (Pit-1) to promote BSP mRNA expression in rat ROS17/2.8 cells.^57^

Platelet-derived growth factor BB (PDGF-BB) promotes BSP mRNA and protein expression in SaOs-2 cells (5 ng/mL, 12 h), by interacting with the CRE1/2, AP1(3) and SSRE1 promoter elements and regulating the transcription factors CREB1, phospho-CREB1, c-Jun, c-Fos, JunD and Fra2.^17^ Insulin-like growth factor 2 (IGF-II) (50 ng/mL, 6 h) enhances BSP gene and protein expression in rat ROS 17/2.8 cells via Tyr kinase, ERK1/2, and PI3K/Akt signalling pathways, and acts on the CRE, FRE and HOX promoter elements.^58^ In contrast, tumour necrosis factor alpha (TNF-α/TNF) (10 ng/mL, 24 h) decreases BSP mRNA expression in these cells through Tyr kinase signalling and by targeting a CRE response element.^59^

#### Other factors in the regulation of bone sialoprotein expression

Calcium hydroxide [Ca(OH)₂], which is involved in the synthesis of HA, enhances BSP gene expression in SaOs-2 cells (0.4 mmol/L, 3 h).^60^ Ca(OH)₂ interacts with the CRE1/2, and FRE elements within the promoter region of human BSP gene, regulating the transcription factors CREB1, c-Jun, c-Fos, JunD, Fra2, Dlx5, Msx2, Runx2 and Smad1. Ca(OH)₂ also targets the co-activator p300.^60^ Similarly, in rat ROS 17/2.8 cells and BMSCs, Ca(OH)₂ (0.4 mmol/L, 6 and 12 h) stimulates BSP mRNA and protein expression via PKC, PKA, PI3K and ERK1/2 signalling pathways and CRE1/2 and FRE promoter elements.^61^ Matrix metalloproteinase 9 (MMP-9) (400 ng/mL) rapidly induces BSP protein in human breast adenocarcinoma (BAC) MDA-MB-231 cells (25 min),^62^ while MMP-2-deficiency increases BSP mRNA in murine BMSCs,^63^ suggesting a suppressive function.

## Expression of bone sialoprotein

The expression of BSP is predominantly confined to mineralised connective tissues. BSP is highly abundant in bone, where it is present in the ECM,^4^ and expressed by osteoblasts,^4,64,65^ osteoclasts,^4^ and osteocytes.^4,64^ BSP is also found in dentin^2^ and chondrocytes.^4^ The ECM of bone consists of an organic component, primarily HA and an inorganic component, largely COL (90%). BSP constitutes ~11%–15% of the non-collagenous proteins in bone,^66,67^ in stark contrast to its much lower levels in dentin, where it accounts for less than 1%.^67^ Beyond bone, BSP has also been detected at low levels in trophoblast cells of the placenta,^4,64^ as well as in macrophages and tubular cells of the kidney.^68^ In the pancreas, BSP expression has been observed in islet, ductal and smooth muscle cells.^69^ Interestingly, BSP is also expressed in (osteotropic) cancer.

### Expression of bone sialoprotein in cancer

#### Expression of bone sialoprotein in primary and secondary cancer tissue and tumour cells

Numerous studies have investigated the protein expression of BSP in primary tumours, metastases and cancer cell lines, using immunohistochemistry (IHC), double immunofluorescence (IF), fluorescence-activated cell sorting (FACS), and western blotting. BSP is expressed in a wide range of cancer cell lines and tumour cells associated with both primary and secondary malignancies. Positive BSP protein expression has been identified in tumour cells from glioma, breast, oral squamous cell, kidney, multiple myeloma, lung, osteosarcoma, ovarian, pancreatic and prostate cancers (Table 1). Tumour cells show heterogenous positive staining, primarily localised in the cytoplasm. Notably, BSP protein expression in primary breast tumour tissues is significantly higher in patients who later develop bone metastases, compared to those without bone metastases.^70^ With the exception of mineralised connective tissues, healthy or benign tissues generally exhibit minimal or absent BSP protein expression.

**Table 1:** Protein expression of bone sialoprotein (BSP) in primary tumours and healthy tissue

Cancer (Subtype)	Protein expression of bone sialoprotein		Quantification	Reference
	Normal (*n*)	Tumour (*n*)		
Bone (OS)	–/o^Matched tissue^	+++* (32)	NA	^90^
Brain (HGG, LGG)	(16)	100% positive* (270) 73% + 27% +++	IHC	^91^
Breast (IDC, ILC)		87.7% positive Heterogenous staining intensity (+/++/+++)	IHC	^70^
Breast	–/o	89% positive (454)	IHC	^89^
Breast (in situ carcinoma, invasive carcinoma)	–/o^Matched tissue^	87% positive* (51) Cytoplasmic	IHC	^78^
Oral mucosa (OSCC)	–	90% positive (87) Cytoplasmic and perinuclear	IHC	^168^
Kidney				
with bone MET, non-bone MET or no MET	Macrophages and tubular cells^Matched tissue^	85% positive (75) Heterogenous staining intensity (+/++/+++)	IHC	^68^
Bone marrow (MM)		100% positive (5) Heterogenous staining intensity (+/++/+++)	Double IF FACS	^72^
Lung (LUSC, LUAD, BaC)	–/o Cartilage	100% positive* (48) Heterogenous staining intensity Cytoplasmic and membrane-bound	IHC	^79^
Ovary		21% positive (38) (++/+++) 79% –/o	IHC	^79^
Pancreas (PDAC)	80% – (10) 20% +/++ Cytoplasmic in islet, ductal and smooth muscle cells	30% positive (20) Diffuse staining of nearby non-target cells	IHC	^69^
Prostate (negative BPH, pAdC)	16% (64) Cytoplasmic and perinuclear	80% positive Cytoplasmic and perinuclear	IHC	^160^
Prostate		100% positive (5) Cytoplasmic with diffuse staining of nearby non-target cells and ECM	IHC	^86^
Prostate	–/o	78.9% positive (180) Heterogenous staining intensity (+/++/+++) Mainly cytoplasmic	IHC	^71^

– undetectable/no expression, o very low expression, + low expression, ++ moderate expression, +++ high expression

*BaC* bronchioalveolar, *BPH* benign prostate hyperplasia, *ECM* extracellular matrix, *FACS* fluorescence-activated cell sorting, *HGG* high-grade glioma, *IF* immunofluorescence, *IDC* invasive ductal carcinoma, *IHC* immunohistochemistry, *ILC* invasive lobular carcinoma, *LGG* low-grade glioma, *LUAD* lung adenocarcinoma, *LUSC* lung squamous cell, *MET* metastasis, *MM* multiple myeloma, *OS* osteosarcoma, *OSCC* oral squamous cell carcinoma, *pAdC* prostatic adenocarcinoma, *PDAC* pancreatic ductal carcinoma, *NA* not available

*P < 0.05 compared to control

Importantly, tumours actively synthesise BSP, with significantly elevated mRNA levels observed in glioma, breast, colon, gastric, kidney, lung, osteosarcoma, pancreatic, rectal and thyroid tumour tissue compared to matched or healthy controls (Table 2). Moderate to strong BSP synthesis is particularly evident in prostate cancer^71^ and multiple myeloma cells.^72^ Table 3 summarises BSP protein expression in human tumorigenic and non-tumorigenic cell lines as assessed by western blotting and FACS.

**Table 2:** mRNA expression of bone sialoprotein (BSP) in primary tumours and healthy tissue

Cancer (Subtype)	mRNA expression of bone sialoprotein		Source (*n*)	Quantification	Reference
	Normal (*n*)	Tumour (*n*)			
Bone (OS)	+	+++* (85)	TARGET database		^90^
Bone (OS)	–/o	+++*	Matched tissue (15)		^90^
Brain					
(HGG) (LGG)	++	+++*^a^ (30) ++* (25)	Patients	RT-PCR	^91^
Brain (GBM)	++	+++^NA^	TCGA		^158^
Breast					
(Ductal) (Lobular)	o	+++* (31) + (31)	Matched tissue (53)	Cancer profiling array	^49^
Breast with bone MET and non-bone MET		+++ (6)	Patients	RNA sequencing	^18^
Breast		+++	GEPIA2 database		^18^
Breast		+/++/+++ (18)	Patients	RT-PCR	^169^
Colon	o	+++*	Matched tissue (39)	Cancer profiling array	^49^
Gastric (stomach)	o	+++*	Matched tissue (27)	Cancer profiling array	^49^
Kidney	o	++*	Matched tissue (21)	Cancer profiling array	^49^
Bone marrow (MM)		++/+++ (5)		In situ hybridisation	^72^
Lung	o	+^NS^	Matched tissue (21)	Cancer profiling array	^49^
Lung					
(LUAD) (LUSC)	o o	+++* +++*	TCGA		^92^
Lung (LUAD)		80% +/++/+++ (10)	Patients	RT-PCR	^79^
Ovary	o	++^NS^	Matched tissue (17)	Cancer profiling array	^49^
Pancreas (PDAC)	41% o 59% – (27)	86% +* (29)	Patients Organ donors	RT-PCR	^69^
Prostate	o	++	Matched tissue (4)	Cancer profiling array	^86^
Prostate	–/o	+++ (8)	Patients	In situ hybridisation	^71^
Rectum	o	+++*	Cancer profiling array		^49^
Thyroid	o	++*	Cancer profiling array		^49^
Uterine (Endometrium)	o	+^NS^	Cancer profiling array		^49^

– undetectable/no expression, o very low expression, + low expression, ++ moderate expression, +++ high expression

*GBM* glioblastoma, *GEPIA2* gene expression profiling interactive analysis 2, *HGG* high-grade glioma, *LGG* low-grade glioma, *LUAD* lung adenocarcinoma, *LUSC* lung squamous cell, *MET* metastasis, *MM* multiple myeloma, *NA* not available, *NS* non-significant, *OS* osteosarcoma, *PDAC* pancreatic ductal carcinoma, *TARGET* therapeutically applicable research to generate effective treatments, *TCGA* the cancer genome atlas

*P < 0.05 compared to control

^a^compared to LGG

**Table 3:** Protein and mRNA expression of bone sialoprotein (BSP) in primary cells and cell lines of healthy and tumourigenic origin

Cell/Tumour Type	Cell (line)	BSP expression		Quantification	Reference
		mRNA	Protein		
Bone Primary osteoblast	hOB	+++		RT-PCR	^90^
Bone cancer					
OS OS	MG-63 SaOs-2	+++ +++		RT-PCR	^13,90^
Bone marrow					
MM MM MM MM MM	L363 LP-1 MMS-1 OPM-1 U266	+/++ +/++ +/++ +/++ +/++	+++ o + ++ +++	In situ hybridisation FACS	^72^
Breast					
Immortalised breast epithelial	MCF-10A		o	Western blot	^131^
Breast cancer					
BAC BAC BDC	MCF-7 MDA-MB-231 T-47D	++ o +	+++	RT-PCR Western blot	^50,131,169^
Lung cancer					
NSCLC NSCLC	A-549 CL1-5		+++ ++	Western blot	^92^
Pancreas	Activated PSCs	o		RT-PCR	^69^
Pancreatic cancer					
PDAC PDAC PDAC PDAC PDAC PDAC PDAC PDAC	ASPC-1 BxPc-3 Capan-1 Colo-357 MIA PaCa-2 Panc-1 SU.86.86 T3M4	+ + – + – – + +		RT-PCR	^69^
Prostate					
Primary epithelial Immortalised epithelial Immortalised BPH	HuPepiC RWPE1 WPE1-NB26	+ –/+ +	– o/– +	RT-PCR Western blot	^55,160^
Prostate cancer					
Derived from LNCaP pAdC pAdC pAdC	C4-2B DU145 LNCaP PC-3	– o –/o/+++ +++	+++ + +	RT-PCR Western blot	^13,55,160,170^

– undetectable/no expression, o very low expression, + low expression, ++ moderate expression, +++ high expression

*BAC* breast adenocarcinoma, *BDC* breast ductal carcinoma, *BPH* benign prostatic hyperplasia, *hOB* human osteoblast, *HuPepiC* human prostate epithelial cell, *MM* multiple myeloma, *NSCLC* non-small cell lung cancer, *OS* osteosarcoma, *pAdC* prostatic adenocarcinoma, *PDAC* pancreatic ductal carcinoma, *PSC* pancreatic stellate cell, *FACS* fluorescence-activated cell sorting

Additionally, in vitro RT-PCR studies typically show low or undetectable BSP mRNA in non-tumorigenic human primary cells and cell lines, whereas tumour cell lines exhibit variable expression depending on tumour type and origin (Table 3). Data remain limited on BSP protein expression in pancreatic cancer and BSP mRNA expression in lung cancer cell lines.

The expression of BSP has also been evaluated in skeletal and non-bone metastases. Metastases to the bone, central nervous system (CNS), adrenal glands, lymph nodes, liver and lungs stain positive for BSP protein. In bone metastases from breast cancer, including invasive ductal carcinoma, moderate to strong BSP protein is detected in 100% (*n* = 42) of tumour cells.^50,73^ In non-small cell lung cancer (NSCLC), BSP protein is present in 20% (6/30) of primary tumours versus 80% (24/30) of bone metastases, and in 31% (8/28) of non-bone metastases [e.g. liver, CNS, adrenal glands].^74^ Similarly, BSP protein is expressed in 100% (*n* = 9) of pancreatic ductal adenocarcinoma (PDAC) lymph node and liver metastases.^69^

However, studies on BSP gene expression in metastases remain sparse. In situ hybridisation using cRNA probes revealed positive, heterogenous BSP staining in all tumour cells from intraductal breast cancer bone metastases (*n* = 10).^50^ Furthermore, BSP mRNA expression is significantly higher in breast cancer bone metastases versus non-bone metastases,^18^ though interpretation is limited by small sample size (*n* = 6), lack of reported *p* values, and unknown group distributions. Using RT-PCR, Kayed et al.^69^ reported weak BSP mRNA expression in pancreatic cancer lymph node metastases (*n* = 18), comparable to expression levels in primary pancreatic tumours (*n* = 11).

Metastases to bone alter physiological bone remodelling: advanced breast and lung cancers predominantly cause osteolytic lesions (excessive bone degradation),^75,76^ while prostate cancer typically induces osteosclerotic lesions (excessive bone formation).^77^ BSP expression in skeletal metastases may modulate both tumour growth and pathological bone remodelling, as explored in this review.

Interestingly, BSP protein is also significantly elevated in breast tumours associated with microcalcifications^78^ and correlates with microcalcifications in lung cancer.^79^ By promoting HA nucleation,^37^ BSP may contribute to microcalcification formation, thereby modifying the tumour microenvironment. This mineral-rich environment may prime tumour cells for colonisation of the bone microenvironment, supporting survival and facilitating bone metastasis,^78^ in line with Paget’s ‘seed and soil’ hypothesis.^80^

##### Conclusions

Research on BSP synthesis in metastases, particularly in bone, is limited. The variability in quantification methods, including RT-PCR, in situ hybridisation and RNA sequencing, complicates precise characterisation of BSP synthesis in secondary cancer tissue or metastatic cancer cells themselves.

#### Expression of bone sialoprotein in the serum

BSP expression has also been quantified the sera of cancer patients, with and without skeletal or non-bone metastases, as well as in healthy individuals. In controls, serum BSP concentrations range from 7.51 ± 1.31 ng/mL to 154 ± 13 ng/mL, displaying high individual variability. In general, studies show significantly elevated serum BSP concentrations in cancer patients compared to controls, with further increases in those with bone or non-bone metastases (Table 4). However, one study^69^ found a trend toward decreased serum BSP in PDAC patients (*n* = 8) versus controls (*n* = 8). Serum BSP concentrations exhibit great variability between studies and experimental groups, possibly due to differences in detection methods. Reports of BSP concentrations above 100 ng/mL employed competitive enzyme-linked immunosorbent assay (ELISA), while lower values (<100 ng/mL) were reported using standard ELISA or radioimmunoassay (RIA). Standard ELISA, which uses two antibodies, provides high specificity; but may underestimate serum concentrations if an epitope is not accessible.^81^ In contrast, competitive ELISA, using a single antibody, is more sensitive but may be susceptible to cross-reactivity.^82^ RIA shares similarities with competitive ELISA but employs radiolabelled antigens for detection.^83^ Overall, the sensitivity, specificity and reliability of BSP quantification were likely influenced by the selected quantitative method.

**Table 4:** Bone sialoprotein (BSP) serum levels collected from cancer patients and healthy controls

Cancer (Subtype)	Mean ± SD (ng/mL)		Quantification	Reference
	Normal (*n*)	Tumour (*n*)		
Bone marrow (MM)	≈10.5 ± 12.97 (133)	≈39.6 ± 26.98* (32)	RIA	^171^
Bone marrow (MM)	≈10.1 ± 6.0 (139)	≈27.6 ± 28.8* (62)	RIA	^84^
Bone marrow (MM)	≈12 ± 2.5 (68)	≈13.4 ± 7.5^NA^ (16)	RIA	^172^
Breast	154 ± 13 (77)	318 ± 18^NA^ (20)	Competitive ELISA	^173^
Breast				
with bone MET	≈10.5 ± 12.97 (133)	≈17.8 ± 21.85* (19)	RIA	^171^
Colon	154 ± 13 (77)	373 ± 19^NA^ (20)	Competitive ELISA	^173^
Lung	154 ± 13 (77)	155 ± 11^NA^ (20)	Competitive ELISA	^173^
Lung (NSCLC)				
with bone MET with non-bone MET	12.33 ± 5.29 (110)	44.61 ± 15.38*^a^ (72) 22.22 ± 13.88* (74)	ELISA	^85^
Pancreas (PDAC)	≈8.0 ± 1.63 (8)	≈6.5 ± 2.58^NA^ (8)	RIA	^69^
Pancreas (PDAC)	≈0.79 ± 1.48 (39)	≈1.72 ± 2.07* (132)	ELISA	^88^
Prostate	114 ± 63 (110)	222 ± 98^NA^ (102)	Competitive ELISA	^86^
Prostate	154 ± 13 (77)	285 ± 19^NA^ (20)	Competitive ELISA	^173^
Prostate				
with bone MET with non-bone MET	7.51 ± 1.31 (42)	38.93 ± 9.12*^a^ 9.23 ± 3.12* (83)	ELISA	^87^

*ELISA* enzyme-linked immunosorbent assay, *PDAC* pancreatic ductal carcinoma, *MM* multiple myeloma, *NA* not available, *NSCLC* non-small cell lung cancer, *RIA* radioimmunoassay

*P < 0.05 compared to control

^a^compared to non-bone metastases (MET)

#### Bone sialoprotein as a potential cancer biomarker

The quantification of BSP in serum shows promise as a cancer biomarker. Elevated serum BSP levels are associated with tumour grade, cancer stage and secondary cancer developments, such as skeletal metastases, in patients with multiple myeloma,^84^ lung,^85^ and prostate cancers.^86,87^ However, one study reported that TNM staging, which incorporates tumour size, lymph node status and metastasis, did not significantly influence serum BSP levels in pancreatic adenocarcinoma patients,^88^ suggesting that serum BSP may not universally correlate with clinicopathological features across all cancer types. Despite this, elevated serum BSP has been associated with poorer survival outcomes in multiple myeloma,^84^ lung,^85^ and pancreatic^88^ cancers.

BSP protein expression in primary tumour tissues from breast^70,73,89^ and lung^74^ is associated with the development of bone metastases and non-bone metastases, including to the lungs and potentially lymph nodes. Furthermore, BSP synthesis in primary tumours of osteosarcoma,^90^ glioma,^91^ breast,^18,49^ colon,^49^ lung,^49,92^ pancreatic,^88^ and rectum^49^ cancers correlates with TNM staging, recurrence-free survival and overall survival (OS), underscoring its broader prognostic significance.

##### Conclusions

BSP, particularly when evaluated alongside other biomarkers such as hormone receptors or prostate-specific antigen (PSA), holds potential to facilitate early cancer detection, guide treatment strategies and provide valuable insights into treatment response and disease progression.

## Functions of bone sialoprotein

### Angiogenesis

Angiogenesis is the formation of new blood vessels from existing ones, primarily supporting wound healing and bone formation (osteogenesis),^93,94^ but also contributing to tumour progression.^95^ In vitro studies demonstrate that recombinant human BSP induces angiogenesis in a 2D co-culture model of human umbilical vein endothelial cells (HUVECs) with human dermal fibroblasts after 6 days [(5 nmol/L (≈0.38 μg/mL)],^96^ and in a spheroid model of HUVECs co-cultured with human primary osteoblasts after 24 h (5 μg/mL).^97^ In ovo, recombinant human BSP [(15 μmol/L (≈1 200 μg/mL)] and recombinant human BSP fragment, containing the RGD sequence [(20 μmol/L and 100 μmol/L (≈132 μg/mL and 660 μg/mL)], stimulate angiogenesis in the chicken chorioallantoic membrane (CAM) assay after 2 days.^98^ Similarly, recombinant human BSP (0.5 μg/mL and 5 μg/mL) immobilised in COL1 induces angiogenesis, as indicated by increased vascular density in the in ovo yolk sac membrane assay, after 3 days.^97^ Notably, the presence of an intact RGD sequence is essential for BSP’s pro-angiogenic activity,^96,98^ as it allows BSP to bind to integrin αvβ3 on the endothelial cell surface, thereby promoting angiogenesis.^98^ Additionally, BSP may enhance angiogenesis by engaging with and activating MMP-2.^96^ BSP is also likely to exert pro-angiogenic effects by regulating endothelial cell functions, including proliferation, adhesion, migration and gene expression.

COL1 gels with immobilised recombinant human BSP (5 μg/mL) upregulate the expression of angiogenesis-related genes in HUVECs, including KDR (VEGF receptor 2), PECAM-1 (platelet-endothelial cell adhesion molecule-1), MCAM (melanoma cell adhesion molecule/CD146) and IGF-1 (insulin-like growth factor 1).^97^ Recombinant human BSP (1 μg/mL and 5 μg/mL) immobilised in COL1 gels also stimulates HUVEC proliferation.^97^ Furthermore, recombinant human BSP promotes the adhesion [(50–100 nmol/L (≈4–8 μg/mL), 2 h], and migration [(50–1 000 nmol/L (≈4–80 μg/mL), 12–18 h] of HUVECs by binding of the RGD motif to αvβ3 integrin, but not αvβ5, on the cell surface.^98^

#### Conclusions

The findings suggest that BSP promotes angiogenesis through RGD-integrin αvβ3 interactions, MMP-2 activation and enhanced expression of pro-angiogenic genes. BSP may couple angiogenesis with osteogenesis, as COL is the primary component of the organic ECM of bone, and BSP is actively involved in bone formation. Nevertheless, the complex mechanisms have to be explored in further studies.

#### Bone remodelling

**Fig. 3:**
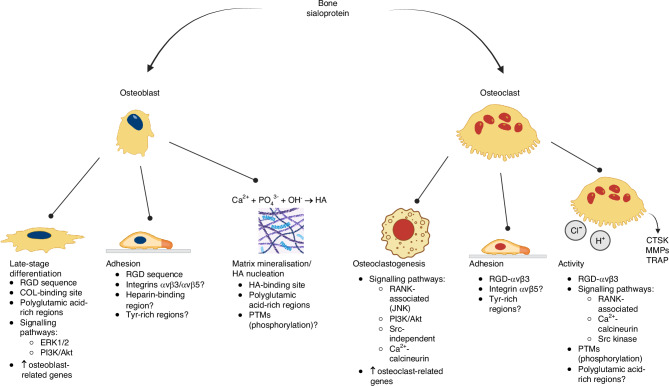
Established and proposed mechanisms of bone sialoprotein (BSP) in osteoblast and osteoclast biology. BSP regulates bone cell function primarily through binding of its arginine–glycine–aspartic acid (RGD) motif to integrin receptors (αvβ3) on the cell surface. BSP-cell interactions activate multiple downstream signalling pathways, including MAPK (ERK1/2), PI3K/Akt, Src-dependent and -independent pathways, calcium–calcineurin signalling and receptor activator of nuclear factor kappa B (RANK)-associated JNK activation, thereby influencing osteoblast and osteoclast differentiation and activity. Additional BSP domains, including the collagen (COL)-binding and hydroxyapatite (HA)-binding motifs, as well as polyglutamic acid-rich regions, contribute to osteoblast differentiation and extracellular matrix (ECM) mineralisation. The heparin-binding motif and tyrosine (Tyr)-rich regions may influence bone cell adhesion. The functional relevance of BSP post-translational modifications (PTMs)—phosphorylation, glycosylation and sulphation—on bone cell biology remains to be fully elucidated. CTSK cathepsin K, MMP matrix metalloproteinase, TRAP tartrate resistant acid phosphatase. Created with BioRender.com

Cytomegalovirus (CMV) BSP-overexpressing mice (8 weeks) show osteopenia, reduced trabecular bone volume and thickness, greater osteoclast numbers, reduced concentrations of osteoblast differentiation markers [alkaline phosphatase (ALP), osteocalcin (OCN)] and elevated concentrations of osteoclast-related markers in the serum [RANKL, tartrate resistant acid phosphatase (TRAP)] than wild-type mice.^99^ Serum calcium is increased, while phosphorus and magnesium are unchanged.^99^ BSP overexpression in mice has no effect on osteoblast surface or number relative to bone surface, potentially due to trabecular bone loss.^99^ Conversely, BSP-deficient mice (4 months) display reduced cortical thickness, increased trabecular bone density, delayed mineralisation and lower relative osteoblast and osteoclast surfaces, indicating low bone turnover.^100^ Thus, BSP plays an important role in bone, as its dysregulation alters bone remodelling. Additional ex vivo and in vitro studies have explored its effects on bone formation, mineralisation, osteoclastogenesis and osteoclast activity. BSP’s dual roles in bone cells and remodelling are depicted in Fig. 3.

#### Osteoblast formation and bone matrix mineralisation

BSP expression is typically absent in early osteoblast precursors,^4,65^ but expressed in mature osteoblasts,^4,65^ suggesting a role in late osteoblast differentiation and mineralisation. BSP-knockout BMSC cultures from 2 to 4-month-old mice show no differences in mesenchymal progenitor pool or early osteoblast differentiation [as indicated by ALP-positive (ALP^+^), unmineralised colonies] compared to wild-type mice.^100^

BSP promotes osteoblast differentiation via ERK1/2 (MAPK) and Akt (PI3K/Akt) signalling,^29^ inducing the expression and activity of osteoblast-related transcription factors [e.g. Runx2, osterix (Osx)]^31^ and osteoblast-related genes (e.g. OCN, ALP, OPN).^29,31,99,100^ BSP-mediated osteoblast differentiation depends on both its RGD motif^31^ and COL-binding region.^29^ BSP also induces primary osteoblast adhesion via its RGD sequence,^65^ suggesting that it anchors osteoblasts to the bone surface and facilitates subsequent matrix mineralisation.

CMV-BSP overexpression enhances primary rat calvarial osteoblast-mediated mineralisation, as demonstrated by an in vitro nodule formation assay (5, 10, 15, 20 and 25 days).^31^ Similarly, osteoblast precursors from 4-day-old CMV-BSP mice calvaria show enhanced bone nodule formation compared to wild-type mice (2 weeks).^99^ In contrast, BSP deficiency impairs mineralisation in primary murine BMSC cultures (18–21 days), as evidenced by fewer mineralised ALP^+^ colonies.^100^ Further experiments using steady-state agarose gel systems demonstrate that porcine BSP nucleates HA at 3 µg/mL, but has low HA-inhibiting activity, as crystal growth is not inhibited at 10 µg/mL.^101^ However, whether human BSP shares similar HA-nucleating capacity as porcine BSP remains unresolved. Findings by Zhu et al.^102^ indicate that BSP expression is dynamically regulated during mineralisation, possibly reflecting changing affinities for COL and HA and supporting distinct stages of bone formation.

##### Conclusions

BSP facilitates osteoblast adhesion to the bone surface, late-stage differentiation and ECM mineralisation through RGD-integrin-mediated signalling (e.g. ERK and PI3K/Akt) and matrix interactions (e.g. COL and HA).

#### Osteoclast differentiation and activity

Osteoclast differentiation: Current evidence indicates that BSP regulates osteoclast differentiation in a concentration- and context-dependent manner, often synergising with RANKL, but occasionally acting independently. Low concentrations (≤50 ng/mL) of human recombinant BSP do not stimulate osteoclastogenesis in murine BMMs or murine monocyte/macrophage-like RAW264.7 cells, regardless of macrophage colony-stimulating factor (M-CSF) (3–7 days).^92,103^ In contrast, higher concentrations (≥100 ng/mL) or overexpression (≈350 ng/mL) promote osteoclast formation in RAW264.7 cells (3 days).^103^ Bovine BSP [30 nmol/L-0.9 μmol/L (≈2.7–81.0 μg/mL)] co-administered with calcitriol (1,25-dihydroxy-vitamin D3) dose-dependently inhibits osteoclastogenesis in co-cultures of murine BMMs and osteoblastic cells (7 days),^32^ suggesting model-specific effects.

BSP enhances RANKL-induced osteoclast differentiation in cell lines and primary cells. Co-treatment of RAW264.7 cells or murine BMMs with BSP and RANKL yields more osteoclasts than RANKL or BSP alone (3–7 days).^92,103^ Spleen and BMSCs cultures from BSP-deficient mice form fewer osteoclasts in response to RANKL (50 ng/mL) than wild-type controls (7 days),^100^ while BMMs from BSP-overexpressing mice treated with RANKL (50 ng/mL) show impaired osteoclast differentiation.^99^ Thus, BSP regulates osteoclastogenesis in a concentration- and context-specific manner, particularly in the presence of RANKL.

Osteoclast activity: BSP alone does not consistently induce bone resorption in osteoclast precursors or mature osteoclasts. Murine BMMs cultured with 50 ng/mL human recombinant BSP fail to form resorption lacunae on osteologic or dentin discs (7 days),^103^ and osteoclasts from BSP-deficient mice display similar resorption pit numbers and areas, as wild-type controls (2–3 days).^100,104^ However, mature osteoclasts from mice or rabbits show dose-dependent resorption increases with bovine [1 nmol/L–1 μmol/L (≈ 0.09–90 μg/mL)] or recombinant human BSP (1–100 ng/mL).^32,103^ RAW264.7 cells treated with 20 ng/mL recombinant human BSP exhibit increased resorption on osteologic discs.^103^ Rabbit osteoclasts treated with 50–100 nmol/L (≈4.5–9.0 μg/mL) bovine BSP form bigger, more numerous resorption pits on ivory slices within 24 h.^32^

BSP synergises with RANKL to enhance osteoclast activity. CMV-BSP BMMs (~350 ng/mL) cultured with 50 ng/mL RANKL generate significantly greater resorption areas on HA or dentin than wild-type cells (5 days).^99^ Similarly, co-treatment with recombinant human BSP (1–100 ng/mL) and murine RANKL (50 ng/mL) enhances pit formation in RAW264.7 and BMMs compared to either treatment alone (4–7 days).^103^ Conversely, in murine BMMs, bovine BSP (5 μg/mL) suppresses PTH-induced calvarial resorption,^43^ suggesting that BSP’s pro-resorptive effects may be RANKL-specific. The PTH source was not specified, though PTH(1-34) promotes osteoclast differentiation and activity via regulation of the RANKL/osteoprotegerin (OPG) axis.^105^

Mechanism of bone sialoprotein-mediated osteoclast differentiation and activity: BSP regulates osteoclast differentiation through integrin-independent signalling. In RAW264.7 cells, BSP induces intracellular calcium fluxes, activating calcineurin and NFATc1 (nuclear factor of activated T-cells, cytoplasmic 1) nuclear translocation.^103^ NFATc1, a key osteoclastogenic transcription factor, subsequently upregulates osteoclast-related genes, including NFATc1, receptor activator of nuclear factor κB (RANK), colony-stimulating factor 1 receptor (c-Fms), TRAP, cathepsin K CTSK, αv and hyaluronan receptor CD44.^103,104^ However, in BSP-deficient mouse spleen-derived osteoclasts or precursors, NFATc1 mRNA is unchanged, suggesting compensatory mechanisms; exogenous recombinant BSP (5 ng/mL) slightly increases NFATc1 expression.^104^ BSP also engages RANK-associated downstream pathways PI3K/Akt and JNK/c-Jun (MAPK),^103^ which are implicated in osteoclast survival^103^ and differentiation.^106,107^ Interestingly, Src inhibition (PP2) does not affect BSP-induced differentiation, indicating Src-independence.^103^

In contrast, BSP-induced resorption is largely integrin αvβ3-dependent. The C-terminal RGD sequence of BSP binds αvβ3, activating Src and triggering cytoskeletal reorganisation. Blocking this interaction using anti-αvβ3 antibodies or RGD peptides (e.g. GRGDS) abolishes BSP-induced resorption in rabbit osteoclasts.^32^ Additional domains, such as N-terminal polyglutamic acid-rich regions, may contribute: a decaglutamyl peptide mimicking this region increases resorption by rabbit osteoclasts,^32^ suggesting partial RGD-independence.^32^ Indeed, BSP’s RGD motif is not required for PTH-induced murine bone resorption.^43^

BSP-integrin engagement induces intracellular calcium signalling via cAMP- and PKC-dependent pathways. In human osteoclast-like GCT23 cells, cAMP analogues and PKC activators suppress BSP-induced calcium influx.^108^ BSP-integrin downstream signalling may induce Src kinase activity and c-Cbl phosphorylation, facilitating osteoclast adhesion and cytoskeletal remodelling. Inhibitors of calcium signalling (BAPTA-AM) or Src (PP2) suppress BSP- and RANKL-induced resorption in RAW264.7 cells,^103^ highlighting their importance in BSP-mediated osteoclast activity.

BSP also regulates osteoclast adhesion and podosome organisation. Although endogenous BSP is not essential for precursor attachment to bone surfaces,^104^ exogenous BSP enhances adhesion in rat osteoclasts^32^ and human GCT23 cells.^108^ BSP-deficient osteoclasts exhibit smaller, more numerous podosomes with impaired motility, indicating reduced migration despite increased turnover.^104^ Thus, BSP may stabilise adhesion structures to support resorption.

PTMs may also modulate BSP’s function. Dephosphorylated bovine BSP shows reduced resorptive activity in mouse BMM-derived osteoclasts, as demonstrated by decreased TRAP activity, COL1-telopeptide (a bone resorption by-product), and matrix degradation compared to native, phosphorylated BSP.^43^ While this underscores the importance of phosphorylation, the relevance to human BSP remains unexamined.

Methodological considerations: Most studies have used murine RAW264.7 cells, BMMs, or rabbit osteoclasts. RAW264.7 cells rapidly differentiate into osteoclasts in response to RANKL alone^109^; notably, M-CSF even suppresses osteoclast formation and activity in these cells when combined with RANKL.^110^ BSP has not been studied in human systems such as THP-1 (human leukaemia monocytic cell line) cells^111^ or primary human osteoclasts,^112^ limiting translational relevance. Furthermore, BSP source (bovine vs. recombinant human), concentration (0.000 1 ng/mL–90 μg/mL) and exposure duration vary widely across studies. Substrate choice may also affect outcomes; mineralised substrates (e.g. dentin, bone) contain native ECM proteins like OPN and BSP, potentially enhancing enzyme expression (e.g. TRAP, CTSK).^113^ Nevertheless, resorption pit formation is generally comparable across natural substrates.^103^

##### Conclusions

In animal models, BSP enhances RANKL-induced osteoclastogenesis via calcium, PI3K/Akt, and JNK signalling and promotes osteoclast activity through αvβ3-mediated activation of Src and cytoskeletal reorganisation. However, its role in human osteoclast differentiation and resorption remains to be elucidated, highlighting the need for studies in human systems.

#### Relationship between bone sialoprotein and osteopontin in bone remodelling

Models of hindlimb disuse (e.g. tail suspension) induce bone loss in BSP-knockout mice,^100^ whereas OPN-knockout mice are protected from such loss,^114^ highlighting a pivotal role for OPN in bone resorption under reduced mechanical loading. Both BSP and OPN facilitate osteoclast-mediated bone resorption by enhancing osteoclast attachment to bone surface via αvβ3 integrins.^32,115^ Following resorption, OPN may suppress new bone formation by regulating BSP’s pro-mineralisation activity, or by directly binding HA to block BSP-HA interactions.^33^ While BSP promotes matrix mineralisation,^27,100^ OPN inhibits it,^116^ indicating opposing roles in bone formation.

In vivo, BSP, but not OPN, is required for cortical bone regeneration, as evidenced by increased osteoid surface and reduced mineral density in BSP-deficient mice.^117^ Interestingly, OPN gene expression is downregulated in 4-month-old BSP-knockout mice^100^ and in osteoclast cultures derived from BSP-deficient spleen cells.^104^ In contrast, BSP mRNA is upregulated in 14-day-old OPN-knockout mice,^117^ suggesting compensatory regulatory mechanisms.

##### Conclusions

BSP and OPN regulate bone remodelling. OPN appears more critical in bone resorption, while BSP may be essential for bone formation and regeneration.

## Bone sialoprotein in cancer progression and immune evasion

**Fig. 4:**
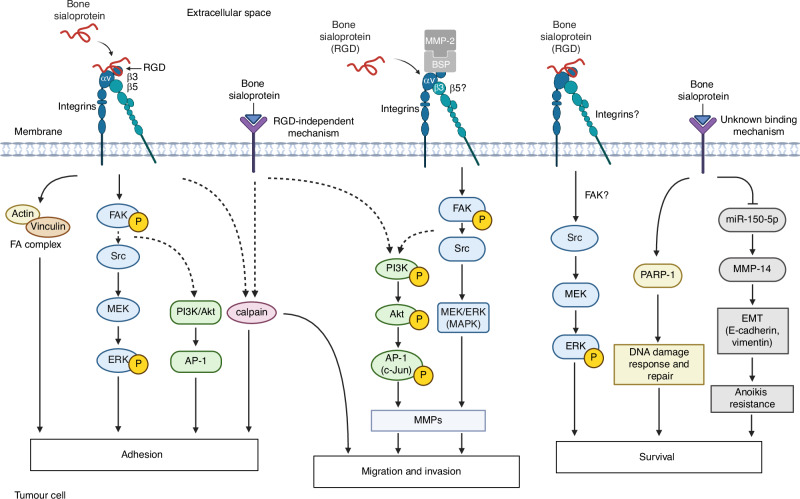
Established and proposed signalling pathways involved in bone sialoprotein (BSP)-mediated tumour cell adhesion, migration, invasion and survival. BSP modulates tumour cell behaviour primarily via binding of its arginine–glycine–aspartic acid (RGD) motif to integrin receptors, predominantly αvβ3 and to a lesser extent the less well-defined αvβ5, on the cell surface. These interactions activate several downstream pathways, including focal adhesion kinase (FAK), Src–MEK/ERK (MAPK), and PI3K/Akt/AP-1 signalling, which together regulate matrix metalloproteinase (MMP) expression, translocation and cancer cell motility. In addition, calpain-mediated signalling—potentially via both RGD-dependent and -independent pathways—promotes tumour cell adhesion, migration and invasion. Activation of Src–MEK/ERK signalling, poly(ADP-Ribose) Polymerase 1 (PARP-1) activity, and the microRNA-150-5p (miR-150-5p)/MMP-14 axis (via mechanisms that remain undefined) further support epithelial–mesenchymal transition (EMT), DNA damage response and repair, and tumour cell survival. The heparin-binding domain and tyrosine (Tyr)-rich regions may contribute to adhesive interactions. The roles of BSP’s post-translational modifications (PTMs)—phosphorylation, glycosylation and sulphation—in regulating tumour cell function remain to be fully elucidated. FA focal adhesion, P phosphorylation. Created with BioRender.com

An overview of signalling pathways through which BSP regulates tumour cell adhesion, migration, invasion and survival is presented in Fig. 4.

### Cancer cell proliferation

Cancer cell proliferation is fundamental to tumour growth, metastasis and aggressiveness, influencing disease progression and treatment response.^118^ The effect of BSP on cancer cell proliferation has been examined using assays such as MTT, Alamar Blue, sulforhodamine B (SRB),^119^ cell counting kit-8 (CCK-8),^120^ and colony formation.^121^ BSP expression has been modified through microRNA (miRNA)-mediated conditional knockdown, small-interfering RNA (siRNA) silencing and CMV-BSP vector overexpression. Additionally, anchorage-based approaches (adhesion to BSP) and exogenous BSP treatments have been used.

Rat BSP and recombinant rat BSP fragments stimulate the proliferation of melanoma (MDA-MB-435 and LCC15-MB) and human BAC (MDA-MB-231) cells [1 μmol/L (≈43.4 μg/mL), 3-5 d].^122^ Conversely, recombinant human BSP has no effect on PDAC (MIA PaCa-2 and Panc-1) cells [0.006 μmol/L (≈0.4 µg/mL), 2 d], but decreases proliferation by ~46% in other PDAC (Capan-1 and SU.86.86) cells under the same conditions, without inducing cytotoxicity.^69^ Recombinant human BSP (100 nmol/L and 200 nmol/L) partially rescues the proliferation of siBSP-transfected MDA-MB-231 cells (50 nmol/L) after 72 h, but not 48 h.^123^ Similarly, BSP overexpression promotes proliferation in osteosarcoma MG-63 cells (2 days),^90^ and BAC (MCF-7 and MDA-MB-231) cells over multiple days (2, 3 and 4 days),^18^ while BSP knockdown reduces proliferation in MCF-7 (2, 3 and 4 days)^18^ and MG-63 cells (2 days).^90^ Silencing BSP significantly inhibits proliferation (2, 3, 4 and 6 days),^18,124^ and colony formation (6 days) in MDA-MB-231 cells.^124^ Interestingly, Sharp et al.^125^ reported no effect of BSP overexpression on MDA-MB-231-BAG cell proliferation in vitro across multiple time-points (4, 6, 8, 10, 12 and 14 days), an MDA-MB-231 derived cell line transduced with the bacterial β-galactosidase (BAG) retroviral vector. In vivo, however, injection of MDA-MB-231-BAG CMV-BSP overexpressing cells into the mammary fat pad or intracardially in nude mice enhanced tumour growth (tumour volume) at both primary and metastatic sites (4–5 weeks).^125^

Mechanistically, BSP-induced proliferation may involve RGD-mediated interactions with integrins αv and αvβ5.^122^ While integrin αvβ3 does not appear essential in BSP-induced proliferation of BAC MDA-MB-231 or melanoma (MDA-MB-435 and LCC15-MB) cells,^122^ it may mediate proliferation in other cancers. Additionally, BSP may promote breast cancer cell proliferation by upregulating SMAD family member 4 (SMAD4) expression^18^ and Poly(ADP-Ribose) Polymerase 1 (PARP-1) activity.^123^ Though typically tumour suppressive,^126^ SMAD4 enhances the proliferation, migration and invasion of breast cancer cells in vitro,^18^ thereby promoting breast cancer progression. PARP-1 regulates cell cycle and DNA repair, facilitating cancer cell proliferation.^127^

#### Conclusions

Although distinct proliferation assays have been used, BSP overexpression generally promotes cancer cell proliferation in vitro and in vivo, while BSP knockdown reduces it. However, most findings are derived from human BAC MDA-MB-231 cells, warranting further research into BSP’s (endogenous and exogenous) effects in other tumour types, such as prostate or lung cancer.

#### Cancer cell survival

Cancer cell survival is crucial for tumour progression and metastasis, enabling resistance to programmed cell death, ECM detachment, intravasation and extravasation, immune evasion, hypoxia adaptation and adaptation and survival in the microenvironment of secondary sites.^128–130^

CMV-BSP expression enhances survival in MDA-MB-231, Hs578t and PC-3 cells following serum withdrawal (1, 2, 3, 4, 5, 6, and 7 days).^38^ In contrast, BSP expression in 2D and 3D (spheroid) cultures of MDA-MB-231 cells is not correlated with apoptosis (7, 14 and 21 days).^131^

The pro-survival effects of BSP require the RGD motif, as transfection of cancer cells with BSP-KAE (Lys-Ala-Glu), where the RGD motif is replaced with KAE, does not promote survival over multiple time points (1, 2, 3, 4, 5, 6 and 7 days).^38^ CMV-BSP expression in MDA-MB-231 cells enhances survival through Src kinase and MEK/ERK (MAPK) signalling (3 days),^38^ potentially activating NF-κB and AP-1 transcription factors and promoting the expression of anti-apoptotic proteins (e.g. Bcl-2 and Mcl-1).^132,133^ However, endogenous BSP expression does not alter Bcl-2 protein levels in siBSP-transfected MDA-MB-231 cells,^123^ but may regulate PARP-1 activity,^123^ thereby supporting cancer cell survival.^134^ Despite these findings, whether endogenous or exogenous BSP exerts anti-apoptotic effects in MDA-MB-231—or other cancer cell types—remains unclear.

Exogenous recombinant BSP enhances anoikis resistance (a form of programmed cell death due to detachment) in NSCLC cells (A-549 and CL1-5) in a dose- [0–30 ng/mL (0–0.3 µg/mL)] and time-dependent (1, 2 and 3 days) manner.^135^ Anoikis-resistant cells exhibit changes in epithelial-mesenchymal transition (EMT) markers, including increased vimentin and decreased E-cadherin, detectable at both transcriptional and translational levels. Findings indicate that BSP promotes EMT-mediated anoikis resistance in NSCLC cells via MMP-14-dependent ERK activation and suppression of microRNA (miR)-150-5p synthesis.^135^ While miR-150-5p typically promotes tumour progression by enhancing survival and suppressing apoptosis,^136^ its role can be context-dependent, with both oncogenic and tumour-suppressive effects.^137^

In vivo, A-549 cells injected into the caudal artery of nude mice show BSP-dependent miR-150-5p downregulation, MMP-14 upregulation, and enhanced anoikis resistance, thereby promoting lung cancer metastasis (8 weeks).^135^

##### Conclusions

Current evidence indicates that BSP enhances breast and prostate cancer cell survival through RGD-integrin MEK/ERK signalling and facilitates anoikis resistance in lung cancer via miR-150-5p/MMP-14, ultimately supporting metastatic colonisation. However, data on the long-term effects of BSP are limited and further research is needed to clarify BSP’s role in survival and apoptosis resistance.

#### Complement evasion by binding to factor H

Activation of the complement cascade leads to complement-mediated cell lysis of pathogens as well as infected or damaged cells, contributing to infection defence and the maintenance of tissue homoeostasis.^138,139^ Factor H, a regulatory glycoprotein of the complement system, suppresses complement activation on host cells by preventing factor B from binding to C3b, thereby inhibiting C3bBb complex formation^138–140^ and subsequent cell lysis.

Recombinant human BSP (10 µg/mL, 2 h) has been shown to initially bind to human multiple myeloma U-266 cells through RGD-integrin αvβ3 interactions.^141^ The interaction facilitates the recruitment and sequestration of factor H, which inhibits complement-mediated cell lysis and enables multiple myeloma cells to evade immune system attack.^141^ Additionally, recombinant human BSP (10 µg/mL, 2 h) has been reported to protect human breast cancer MCF-7 cells from complement attack.^141^ Notably, however, Fedarko et al.^141^ did not investigate the involvement of receptor CD44 or other RGD-binding integrins, such as integrin αvβ5, in BSP-mediated complement evasion.

Based on some of the reported functional similarities within the SIBLING protein family, BSP may, like OPN, have a role in immune modulation. OPN promotes the expansion of immunosuppressive cells such as myeloid-derived suppressor cells (MDSCs),^142^ suppressing T-cell and natural killer (NK) cell activity. Moreover, OPN has been shown to inhibit human CD8^+^ T cell activation and proliferation in vitro,^143^ thereby reducing T-cell mediated cytotoxicity and cancer cell death. Whether BSP exerts similar effects remains unknown.

##### Conclusions

The data indicates that BSP stimulates immune evasion and survival of circulating metastatic cells via sequential binding to integrins and factor H. Further research is warranted to support and clarify BSP’s immunological roles in cancer progression.

#### Cancer cell adhesion

Cell adhesion refers to the adhesion of cells to each other (cell-cell adhesion) or to their substratum, the ECM (cell-matrix adhesion). Anchorage-dependent cell adhesion is important for tissue integrity, repair and function,^144,145^ but also for cell motility,^146^ such as in cancer.

Plates coated with isolated rat BSP promote the adhesion of triple-negative breast cancer (TNBC) (MDA-MB-231 and Hs578t), human epidermal growth factor receptor 2 (HER2)-positive breast cancer SK-BR-3, and rat osteosarcoma UMR-106 cells (0.3 μg/mL and 0.5 μg/mL, 40 min–1 h).^147^ Melanoma cells (M21, MDA-MB-435, LCC15-MB) also adhere to rat BSP at 0.3 μg/mL and 0.5 μg/mL (40 min–1 h)^147^ or 1 μmol/L (≈43.43 μg/mL, 1 h).^122^ Recombinant rat protein (20 μg/mL, 1 h) induces MDA-MB-231, Hs578t and pAdC PC-3 cell attachment.^38^

Research on human BSP in anchorage-dependent cell adhesion is scarce. Plates coated with recombinant human BSP fragment promote the adhesion of BAC (MDA-MB-231), and melanoma (MDA-MB-435 and LCC15-MB) cells [1 μmol/L (≈6.6 μg/mL), 1 h].^122^ MDA-MB-231 cells bind dose-dependently [0.01-10 μmol/L (≈0.6-676 μg/mL)] to linear and cyclic human BSP peptides (3 h), with cyclic peptides exhibiting stronger adhesive properties.^39^ Recombinant human BSP (0-10 µg/mL, 2 h) mediates dose-dependent adhesion in BAC (MDA-MB-231), pAdC (PC-3) and large-cell lung carcinoma (NCI-H460) cells,^148^ but has no effect on PDAC (Capan-1 and SU.86.86) cells at 6 nmol/L (≈0.4 µg/mL, 2 days).^69^ Additionally, endogenous BSP expression enhances adhesion, as BSP-transduced MDA-MB-231 cells show stronger adhesion relative to vector controls.^38^

Mechanistically, cell adhesion to BSP primarily occurs via its RGD sequence, which engages integrin receptors.^148^ This interaction is BSP-specific: recombinant human BSP-KAE fragments and fibronectin (FN)-derived GRGDSP fail to stimulate MDA-MB-231 adhesion.^122^ Notably, exogenous FN-derived GRGDS has no effect on MDA-MB-231 cell adhesion to human bone ECM, while fragments of recombinant human BSP significantly decrease breast cancer cell attachment to human ECM [2 μmol/L (≈2.4-134 μg/mL)], following 3 h,^39^ highlighting the unique role of BSP in cancer cell adhesion, particularly to bone. BSP’s RGD motif binds different αv-integrins depending on the cancer cell type. Rat BSP and human BSP-derived RGD peptides primarily bind to β3 and, to a lesser extent, αvβ5 on M21 cells^147^; αv, αvβ5, and possibly αvβ3 on LCC15-MB cells^122^; and αv,^122^ β3,^147^ and αvβ5^122,147^ on MDA-MB-435 cells. Rat BSP and recombinant human BSP fragments also bind αvβ5—but not β3—on SK-BR-3 cells^147^; and αv and αvβ5—but not αvβ3—on MDA-MB-231 cells.^122^ Recombinant human BSP binds αvβ5 on MDA-MB-231; αvβ3 and, to a small degree, αvβ5 on PC-3; and primarily αvβ5 and, to a lesser extent, αvβ3 on NCI-H460 cells.^148^

The heparin-binding (LHRRVKI) and COL-binding motifs near the N-terminus of BSP may also regulate adhesion. The rat heparin-binding domain (FHRRIKA) synergises with RGD to promote rat osteoblast-like cell adhesion (FHRRIKA:RGD ratios 75:25, 25:75, 50:50, 4 h), but is insufficient to induce vinculin contact formation and actin cytoskeletal reorganisation alone.^28^ Rat BSP (50 μg/mL, 2 h) binding to COL1 does not increase mouse osteoblast-like MC3T3-E1 cell adhesion versus BSP-coated plastic (50 μg/mL, 2 h),^149^ suggesting that the heparin- but not COL-binding domain facilitates RGD-mediated adhesion. Whether these domains function similarly in humans is not yet determined.

BSP binding activates RGD- and integrin-dependent signalling pathways, inducing focal adhesion complex formation involving vinculin and actin.^38^ In MDA-MB-231, Hs578t and PC-3 cells, RGD-mediated binding stimulates focal adhesion kinase (FAK) and ERK phosphorylation.^38^ Cell-BSP interactions, potentially via integrins, also activate Src-kinase dependent MEK/ERK (MAPK) signalling and AP-1 transcriptional activity.^38^ Conversely, M21 melanoma cell adhesion to BSP is MEK/ERK-independent, as shown using the MEK1/2 inhibitor PD98059 (50 μmol/L, 30 min),^147^ but involves PI3K/Akt and calpain signalling.^147^ Calpain, a calcium-dependent protease family, regulates dynamic cell-ECM interactions.^150^

BSP contains glycosylation sites in its amino-terminal and central domains, and phosphorylation and Tyr sulphation sites in its carboxyl-terminal region, all of which may modulate cancer cell adhesion. Desialylation may enhance BSP’s affinity for COL and, consequently, increase cancer cell adhesion the bone matrix.^41^ Hypophosphorylated or unphosphorylated Thr residues may promote cancer cell adhesion,^42^ as evidenced by increased adhesion of MDA-MB-435 melanoma cells to low-phosphorylated OPN relative to highly phosphorylated OPN.^151^ This may reflect altered integrin affinity (αvβ3 or αvβ5),^152^ affecting RGD-integrin interactions. Glycosylation and phosphorylation may thus suppress BSP-mediated adhesion, while sulphation of Tyr297 or Tyr298^22^ may induce conformational changes which expose the RGD motif and promote integrin binding.^153^

##### Conclusions

BSP enhances cancer cell adhesion through RGD-integrin interactions, activating (Src)-MEK/ERK, PI3K/Akt and calpain pathways, with MEK/ERK signalling involvement potentially varying by cell type. Effects on human osteosarcoma, kidney and thyroid cancer cells remain unknown, and the integrins mediating osteosarcoma and prostate cancer adhesion are yet to be identified. BSP may promote integrin-independent adhesion via the heparin-binding domain.

#### Cancer cell migration and invasion

Cell motility is essential in embryogenesis^154^ and tissue regeneration, such as wound healing.^155^ However, its dysregulation facilitates tumour growth and metastasis.^146,156^ Although migration and invasion are often used interchangeably, we distinguish between them: migration is the movement of cells without traversing a membrane (e.g. COLIV or Matrigel), whereas invasion involves the (active) migration of cells through such barriers.

To investigate BSP’s impact on cancer cell motility, studies have performed scratch assays, transwell migration, matrigel invasion, 3D spheroid invasion and matrigel outgrowth and plug assays. Rat BSP stimulates migration of melanoma M21 (5 μg/mL, 5–8 h),^147^ BAC MDA-MB-231 (20 μg/mL, 8 h),^38^ and UMR-106 rat osteosarcoma cells (0.5 μg/mL and 1 μg/mL, 5–8 h).^147^ Recombinant BSP (0.03 μg/mL, 18 h) stimulates NSCLC A-549 and CL1-5 migration,^92^ while lower concentrations (0.005 μg/mL and 0.01 μg/mL) are ineffective.

Recombinant human BSP stimulates invasion in melanoma (MDA-MB-435S), BAC (MDA-MB-231, MCF-7), thyroid (SW-579) and NSCLC NCI-H520 at 0–100 nmol/L (≈0–7.5 μg/mL, 6–24 h),^157^ and in A-549 and CL1-5 at 0.03 μg/mL (recombinant BSP of unspecified origin, 18 h).^92^ Similar effects occur in pAdC (PC-3, DU-145) [0–100 nmol/L (≈0–7.5 μg/mL), 6–24 h],^157^ but not in pAdC LNCaP or osteosarcoma (SK-ES-1, SaOS-2 and MG-63).^157^ Notably, BSP [6 nmol/L (≈0.405 µg/mL), 24 h] decreases invasion by 13% in Capan-1 and 59% in SU.86.86 PDAC cells.^69^ In 3D cultures, BSP promotes glioblastoma spheroid invasion (9 days).^158^

Endogenous BSP levels also influence cancer cell motility: Overexpression stimulates the migration (8 h, 24 h, 48 h) and invasion (8 h, 24 h, ≤10 days) in BAC (MDA-MB-231, MDA-MB-231-BAG, MCF-7, Hs578t) cells^18,38,125^ and promotes PC-3 invasion (8 h).^38^ Conversely, BSP knockdown through siRNA and miRNA inhibits migration (24 h, 48 h)^18,123,124^ and invasion (24 h) in MDA-MB-231 and MCF-7.^18^ Short hairpin RNA (shRNA)-mediated BSP knockdown reduces NSCLC (A-549 and CL1-5) migration and invasion by ≥50% (18 h).^92^

BSP’s RGD sequence binds to integrins αv and αvβ3 on cancers cells to navigate cell migration and invasion.^38,122,147,157,158^ Similar to BSP-mediated adhesion, the RGD motif appears to be BSP-specific or requires full-length BSP, as recombinant human BSP induces greater encapsulated glioblastoma spheroid invasion than an RGD peptide.^158^ However, this RGD-dependence is partial, since a KAE-substituted recombinant human BSP fragment promotes breast cancer invasion similarly to its RGD-containing counterpart.^122^

RGD-integrin engagement may promote ECM degradation via stimulation of MMP translocation to the cell membrane,^159^ thereby promoting cancer cell migration and invasion. BSP and MMP-2 are co-expressed in prostate neoplasm,^160^ papillary thyroid carcinoma cells,^157^ and their expression significantly correlates in breast and colon cancers.^161^ In human thyroid cancer SW-579 cells, recombinant human BSP forms a complex with αvβ3 and MMP-2 to promote invasion.^157^ Similarly, in NSCLC (A-549, CL1-5), BSP-induced motility requires MMP-14, as MMP-14 silencing or inhibition suppresses migration and invasion.^92^ BSP also influences MMP synthesis: BSP overexpression upregulates MMP-2, MMP-9 and MMP-14 expression in MDA-MB-231, Hs578t and PC-3 cells (24 h).^38^ Treatment with exogenous BSP (30 ng/mL) induces MMP-14 gene and protein expression in NSCLC (A-549, CL1-5) cells, but does not alter MMP-2, MMP-3, MMP-9, or MMP-13 gene expression.^92^ Notably, BSP-transfected MDA-MB-231-BAG cells show no increase in MMP-2 and MMP-9 expression.^125^ Thus, BSP may modulate MMP translocation and/or synthesis in a cell-type-specific manner to facilitate cancer cell motility.

BSP activates several signalling pathways driving cancer cell motility. In M21 cells, migration to BSP is largely mediated by PI3K/Akt and calpain pathways,^147^ where calpain-2 may enhance migration through FAK proteolytic degradation.^162^ In NSCLC cells (A-549 and CL1-5), BSP stimulates PI3K/Akt/AP-1 signalling, inducing phosphorylation of PI3K, Akt and c-Jun to promote AP-1 transcriptional activation, migration and invasion.^92^ In BAC MDA-MB-231 cells, pharmacological inhibition of Src (PP2), MEK (PD98059), or FAK (FRNK) reduces BSP-mediated invasion, implicating FAK/Src and MEK/ERK signalling.^38^ MEK/ERK signalling also contributes to BSP-induced migration in M21 cells.^147^ BSP may further enhance motility and metastasis through EMT stimulation^18^ and activation of the miR-150-5p/MMP-14 axis.^92,135^

In addition, PTMs such as glycosylation and phosphorylation may inhibit BSP-mediated adhesion,^41,42^ yet facilitate cancer cell motility. For example, highly phosphorylated, but not low-phosphorylated, OPN promotes JAR choriocarcinoma cell migration via PI3K/mTOR/p70S6K signalling.^163^ These findings raise the possibility that higher phosphorylation levels of BSP could similarly enhance migration, although this remains to be investigated.

##### Conclusions

While research on BSP in cancer motility is limited, FAK, PI3K/Akt, MEK/ERK (MAPK) and calpain signalling mediate its pro-migratory and invasive effects, potentially in a cell-type-dependent manner. Further studies should clarify exogenous BSP’s effects and mechanisms, particularly in prostate cancer.

**Fig. 5:**
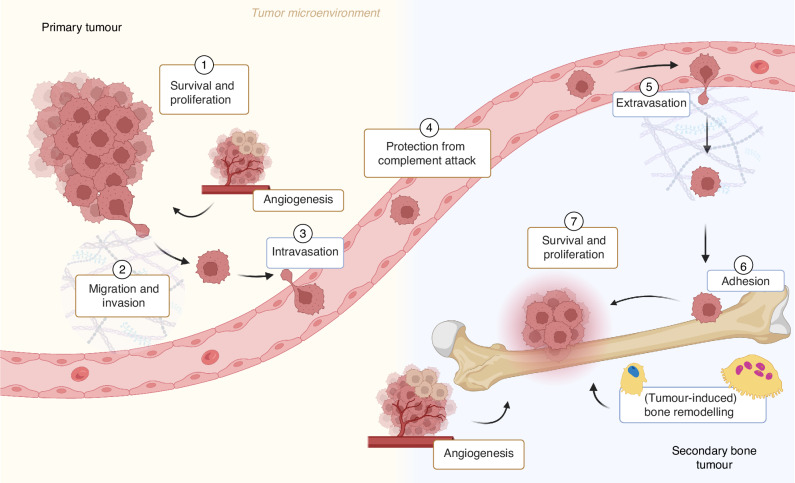
The proposed roles of bone sialoprotein (BSP) in tumour progression and skeletal metastasis development. In the tumour microenvironment, BSP promotes cancer cell proliferation, potentially through arginine–glycine–aspartic acid (RGD)-integrin interactions and by stimulating the expression of SMAD4 and poly(ADP-ribose) polymerase 1 (PARP-1). BSP enhances cancer cell survival via RGD-integrin-mediated MEK/ERK signalling and supports anoikis resistance through the microRNA-150-5p (miR-150-5p)/matrix metalloproteinase (MMP)-14 axis. These effects on survival and epithelial-mesenchymal transition (EMT) enable cancer cell dissemination, migration and invasion, facilitating the establishment of metastases at secondary sites, particularly in bone. Upon entry into the bloodstream, BSP protects disseminating tumour cells (DTCs) from complement-mediated destruction. This occurs through its initial binding to integrin receptors on DTCs, followed by interaction with factor H to block complement activation and suppress cell lysis, further enhancing cancer cell survival and dissemination. At secondary sites such as bone, BSP supports extravasation and tumour colonisation by promoting adhesion, proliferation, survival, migration and invasion of cancer cells. Both endogenous and exogenous BSP expression contribute to cancer cell adhesion and motility via RGD-integrin binding, which activates signalling pathways including focal adhesion kinase (FAK), Src-MEK/ERK (MAPK), PI3K/Akt and calpain-mediated cascades. Once tumour cells colonise the bone, they drive bone remodelling. BSP expression by both tumour and bone cells enhances osteoblast formation, matrix mineralisation, osteoclast differentiation and activity. The extent of bone formation and resorption varies depending on the primary tumour type and individual patient characteristics. Created with BioRender.com

An overview of BSP’s proposed roles in tumour progression, metastasis and (tumour-induced) bone remodelling is shown in Fig. 5.

## Bone sialoprotein as a therapeutic target

Owing to its pro-oncogenic, pro-angiogenic functions and its role in bone remodelling, BSP is an attractive target for therapeutic intervention in cancer and skeletal disease. RNA interference approaches, such as shRNA, siRNA, antisense oligonucleotides (ASOs), or miRNAs, that downregulate BSP expression have been shown to inhibit tumour progression and metastasis. For instance, injection of BSP-silenced NSCLC A-549 cells into nude mice reduces lung metastases compared to controls.^135^ Similarly, BSP knockdown in A-549 or BAC MDA-MB-231 cells decreases tumour burden and osteolytic lesions in bone metastasis models.^92,124,164^

Antibody-based BSP inhibition may provide an alternative by blocking tumour cell adhesion to mineralised bone and modulating bone homoeostasis. However, further studies are needed to evaluate their broader effects on migration, osteoclast activation and in vivo tumour progression.

Another strategy targets the RGD–integrin axis, a key mechanism of BSP-mediated adhesion and signalling. Yet, as this motif also mediates physiological angiogenesis and bone remodelling, agents against it or its integrin receptors (e.g. αvβ3 and αvβ5) require careful design to minimise off-target effects. A Phase II clinical trial (NCT01360840) of the pan-αv integrin inhibitor Abituzumab combined with hormone therapy in prostate cancer patients with bone metastases showed reduced skeletal tumour growth but no benefit in progression-free survival.^165^ Further trials are needed to assess its efficacy in other solid tumours, such as advanced breast or lung cancers, which frequently cause osteolytic lesions.

### Gaps in the field

Despite valuable insights from animal and in vitro studies, major gaps remain in our understanding of BSP. Human data on BSP expression, genetic variation and biomarker potential are limited, partly due to the lack of standardised assays. Species differences and redundancy among SIBLING proteins complicate the interpretation of BSP’s unique functions. Its precise roles in mineralisation, adhesion and signalling within the human bone microenvironment remain unclear. Furthermore, BSP’s involvement in cancer metastasis and implant integration is underexplored. The absence of human-relevant models continues to limit translation, highlighting the need for improved clinical studies and mechanistic research.

## Conclusions and outlook

Collectively, the current literature highlights BSP as a multifaceted protein involved in tumour progression, metastasis development and bone remodelling. BSP is frequently detected at the gene and protein levels in primary tumour tissues across various cancer types. However, studies examining BSP expression in bone metastases remain limited, particularly regarding its mRNA levels in secondary tumours.

BSP’s RGD-mediated binding to integrins is a critical mediator of tumour growth and survival, angiogenesis, cancer cell immune evasion, adhesion and motility, potentially facilitating bone metastasis and anchorage to the bone ECM. However, the specific contributions of other BSP motifs, such as the heparin-binding and COL-binding sequences, to human BSP-driven cancer cell adhesion remain poorly understood. The integrin/BSP/MMP axis and the impact of full or partial EMT in BSP-regulated cancer cell migration and invasion are still insufficiently explored. It remains to be determined whether BSP not only facilitates tumour cell evasion of complement activation but also, for instance, affects the recruitment or expansion of immunosuppressive cells or directly suppresses immune cell activity. The complexity of BSP in tumour progression, its associated signalling pathways (e.g. integrin-dependent and integrin-independent), and its involvement with (pro)MMPs may reflect the heterogeneity of primary tumour types. Notably, current knowledge on BSP-regulated tumour progression and metastasis is largely based on studies using various human cancer cell lines, including melanoma, breast, prostate, lung and pancreatic cancers. To date, the potential role of BSP in kidney or thyroid cancer has received little attention.

Beyond tumour biology, BSP plays a dual role in bone remodelling, promoting both bone formation and resorption. Similar to its function in tumour cells, BSP’s RGD sequence is important for osteoblast adhesion and the activity of osteoclasts. While extensive research has explored its ability to promote osteoblast differentiation and HA nucleation, few studies have examined the signalling pathways involved in BSP-induced late-state osteoblast differentiation. Evidence suggests that BSP synergises with RANKL to facilitate both the activation and resorption phases of bone remodelling. However, how BSP initiates calcium signalling in osteoclast precursors to induce osteoclastogenesis remains unclear. Furthermore, existing studies rely on animal models, leaving BSP’s role in human osteoclast differentiation and resorption activity unresolved. Notably, research on the PTMs of human BSP—such as glycosylation, phosphorylation and sulphation—and their impact on BSP activity remains scarce. This is surprising, given that BSP is a heavily post-translationally modified protein.

Thus, BSP may regulate tumour pathophysiology, osteoclast-mediated bone resorption and osteoblast-driven bone formation, collectively contributing to secondary bone tumour development and associated bone remodelling processes. Unravelling the mechanisms behind BSP’s roles in tumour progression and bone degradation—both physiological and pathological—could lead to improved strategies for detecting, preventing and treating cancer and bone diseases such as osteoporosis or Paget’s disease. Research may contribute to the validation of BSP as a tumour biomarker and support the development, characterisation and preclinical evaluation of BSP-targeting inhibitors or antibodies in in vitro and in vivo experiments (e.g. ovariectomised or tumour xenograft model), ultimately aiming for clinical translation.

Given the limitations of current cancer and bone remodelling therapies—such as the lack of food and drug administration (FDA)-approved drugs for integrins αvβ3 and αvβ5, and the prolonged effects of bisphosphonates even after discontinuation^166,167^—there is an urgent need for novel treatments to enhance patient survival and quality of life (QOL).
